# The online language of work-personal conflict

**DOI:** 10.1038/s41598-023-48193-3

**Published:** 2023-11-29

**Authors:** Gloria Liou, Juhi Mittal, Neil K. R. Sehgal, Louis Tay, Lyle Ungar, Sharath Chandra Guntuku

**Affiliations:** 1 Purdue University, West Lafayette, USA; 2 Rose-Hulman Institute of Technology, Terre Haute, USA; 3 University of Pennsylvania, Philadelphia, USA

**Keywords:** Psychology, Human behaviour

## Abstract

With the blurring of boundaries in this digital age, there is increasing concern around work-personal conflict. Assessing and tracking work-personal conflict is critical as it not only affects individual workers but is also a vital measure among broader well-being and economic indices. This inductive study examines the extent to which work-personal conflict corresponds to individuals’ language use on social media. We apply an open-vocabulary analysis to the posts of 2810 Facebook users who also completed a survey for an established work-personal conflict scale. It was found that the language-based model can predict personal-to-work conflict (*r* = 0.23) and work-to-personal conflict (*r* = 0.15) and provide important insights into such conflicts. Specifically, we found that high personal-to-work conflict was associated with netspeak and swearing, while low personal-to-work conflict was associated with language about work and positivity. We found that high work-to-personal conflict was associated with negative emotion and negative tone, while low work-to-personal conflict was associated with positive emotion and language about birthdays.

## Introduction

With the technology and communication tools available today, the boundary between work and life outside of work has become increasingly blurred, exacerbating issues around work-personal conflict. A recent study found that 55% of workers send digital communication (i.e., texts, calls, emails) to colleagues after working hours, and 30% send digital communication on weekends—while expecting a same-day response^1^. Work-nonwork interference occurs in the other direction as well. More than 60% of employees visit non-work-related websites at work, with more than half admitting to constantly surfing the Internet on the job^2^. Additionally, 60% of online purchases and 70% of Internet porn traffic occur during the 9-to-5 workday^2,3^.

While many studies have looked at work-nonwork conflict, the vast majority of these have considered family as the only or primary part of life outside of work^4–9^. As we continue to examine work-nonwork conflict, researchers need to consider the personal domain (e.g., friends, hobbies, travel). This is an area that has not been given enough attention in the field, especially considering the increasing number of single and/or childless workers^10–12^, whose focus outside of work is likely on personal pursuits.

In this digital age, many work and personal pursuits are shared online. There is a growing repository of information on the lives of workers on social media, and analyzing social media language data is an inexpensive, non-intrusive, and naturalistic method^13^. This study draws on organic social media data to examine work-personal conflict in 2810 participants who completed a survey for an established work-personal conflict scale^9^ and volunteered to share their Facebook posts for language analysis. Answering the call for more inductive approaches in organizational research^14^, we were able to create a language model that can predict work-personal conflict based on social media language and gather detailed insights about work-personal conflict and language use.

### Open-vocabulary approaches to language analysis of social media data

In the past, language analysis used in organizational and psychological studies typically relied on closed-vocabulary methods^15^, which depended on a predefined list of words and phrases. Open-vocabulary language analysis, on the other hand, does not rely on predetermined information but instead employs computational linguistic techniques to produce a collection of language features by extracting single words, multiword phrases, and *topics*—clusters of semantically related words—as well as their relative usages. Because open-vocabulary methods do not rely on a predefined list of words and phrases, they can account for unexpected words and phrases^16,17^ and unconventional language, such as emoticons, slang, and new or uncommon words^13,18^.

Open-vocabulary approaches to language analysis are becoming more popular in many disciplines, and the organizational field is no exception. There have been a variety of studies using open-vocabulary language analysis on social media data to investigate workplace constructs, including job satisfaction^19^, employee engagement^20,21^, workplace culture^22^, reputation^23^, and affect^24,25^. These studies have shown that open-vocabulary social media methods can provide insights that are challenging to assess with survey data or closed-vocabulary methods. For example, Saha et al. investigated the relationship between language use and two facets of job satisfaction—pay and supervision—by analyzing a Twitter dataset of 1.5 million posts^19^. The study found that women expressed greater pay satisfaction than men, and racial minorities expressed greater pay and supervision satisfaction than racial majorities, providing evidence for the “job satisfaction paradox”^26–28^, where disadvantaged groups feel more satisfied than privileged groups. Saha et al. also uncovered linguistic differences between racial groups, finding that racial minorities talked more about basic livelihood while racial majorities talked more about self-actualization. These organic insights about job satisfaction showcase the benefit of using open-vocabulary social media language methods in organizational research.

### Work-personal conflict

There is tremendous value in considering how the work and personal domains interact. The personal domain exhibits different attributes than the work and family domains, including higher levels of choice and lower costs for noncompliance, so examining the personal domain can enable a more thorough understanding of workers’ interrole relationships and conflicts^9,29,30^. The emphasis on choice in the personal domain also makes work-personal conflict an excellent candidate for studying individual-level outcomes (e.g., satisfaction with the need for relatedness). Additionally, there is evidence from previous research that work-personal conflict can help explain the variance in work outcomes (e.g., job performance), outcomes at home (e.g., relationship satisfaction), and health outcomes (e.g., depression, anxiety) that cannot be explained by work-family conflict alone^9^.

Social media data is a sensible option for investigating work-personal conflict. In addition to previous successes with organizational topics^19–22,31^, social media is largely used for personal purposes, with finding and/or maintaining friends as the primary reason cited for using social media^32^. Research also shows that some individuals use social media to write about friendships, health, and political views^33,34^, all of which fall under the personal domain. While both work-to-personal and personal-to-work conflict involve the same two domains, there is evidence from previous work that social media data may not predict them equally well. Specifically, Wilson and Baumann found that personal-to-work conflict was related to satisfaction with the need for relatedness, while work-to-personal conflict was not^9^. Given that social media is used by many to satisfy the need for relatedness^32,35,36^, there may be differences in whether and how personal-to-work conflict and work-to-personal conflict manifest in social media language.

### The present study

The present study utilizes an established work-personal-family scale^9^ that was created for researchers to examine how interrole conflict impacts workers. In this study, we examined whether work-personal conflict can be predicted by language use in Facebook posts. This was accomplished by building and validating a language model that can predict work-personal conflict based on what individuals post on Facebook. Through our analysis, we were also able to identify the topics and topic themes that were prevalent among high conflict and low conflict individuals, providing insights into the impact of work-personal conflict on our daily lives.

Through this study, we aim to fill the following research gaps. First, we investigate whether work-personal conflict can be predicted by online language similar to other individual difference constructs, which is something that has not been studied before. Previous research has shown that individuals’ self-reports of personality (*r* = 0.21–0.42), life satisfaction (*r* = 0.14–0.19), and stress (*r* = 0.12–0.21) can be predicted by social media language^13,16,37,38^, and we expect that work-personal conflict can be similarly predicted. [We searched for individual difference work constructs such as job satisfaction but could not find studies that analyzed predictive accuracy using individuals’ self-reports and Pearson’s r correlation. Saha et al. used Area Under the Curve and Hickman et al. used Mean Cross-Validation and was not investigating predictive accuracy of self-reports^19,39^.] At the same time, the strength of this effect may be moderated by the direction of the conflict, with personal-to-work conflict being more predictable than work-to-personal conflict. Second, there are no previous studies that have examined the differences in social media language use between individuals with high and low work-personal conflict.

Our study adds to the important conversation around the conflicts faced by workers today. More broadly, we believe that assessing and tracking work-personal conflict is particularly compelling as similar work constructs have been shown to impact not only individuals’ experiences but also broader sets of well-being and economic indices^39,40^. Establishing the ability to index and track work-personal conflict through social media data will enable organizational researchers to contribute to related policy and societal discussions.

## Method

### Participants

We recruited participants using Qualtrics, and participants received a small incentive for their participation. All participants provided their written informed consent and agreed to the anonymous use of their survey responses and Facebook posts for research purposes. This study received approval from the Institutional Review Board of Purdue University, and all methods were performed in accordance with relevant guidelines and regulations.

Our analytic sample (*N* = 2810) was a subset of the participants who completed the Qualtrics survey. All participants in the analytic sample were of legal age (18 +), identified as man or woman, currently lived in the United States, were currently employed, and had a Facebook account. We limited our analytic sample to users who wrote at least 500 words across their Facebook status posts in English, removing three participants who only had non-English posts. We also removed the 700,000 non-English posts identified by *langid*, an off-the-shelf language identification tool^41^, from the original total of 4 million posts, leaving 3.3 million posts for analysis. Participants in our study wrote an average of 27,401 words across these posts (median = 11,901; *SD* = 52,568). In our analytic sample, the mean participant age was 43.42 (median = 43; *SD* = 12), and over half (69.62%) were women.

### Work-personal conflict measure

All participants completed measures of work-personal-family conflict as defined by Wilson and Baumann’s work-personal-family scale^9^. The scale consists of 20 questions and measures four directions of conflict: work interfering with personal (work-to-personal; e.g., “The demands of my work interfere with my personal activities.”), personal interfering with work (personal-to-work; e.g., “My personal activities produce stress that makes it difficult to concentrate at work.”), family interfering with personal (family-to-personal; e.g., “The amount of time my family takes up makes it difficult to fulfill personal interests.”), and personal interfering with family (personal-to-family; e.g., “My personal interests prevent me from completing family responsibilities.”). For our study, we focused on two subscales, work-to-personal (alpha = 0.89) and personal-to-work (alpha = 0.90).

We used confirmatory factor analysis (CFA) to test the fit of the measurement model. The model provided a good fit to the data for both work-to-personal and personal-to-work subscales. For work-to-personal conflict: χ^2^ = 91.90, *df* = 5, *p* < 0.001; CFI = 0.99, TLI = 0.98, RMSEA = 0.08, SRMR = 0.02. For personal-to-work conflict: χ^2^ = 29.99, *df* = 5, *p* < 0.001; CFI = 1.00, TLI = 0.99, RMSEA = 0.04, SRMR = 0.01.

### Linguistic analyses

#### Tokenization

We split Facebook posts into words, punctuation, and emoticons using the *happierfuntokenizing* tokenizer^42^. Words that were used by less than 1% of users were not included in the analysis as a way to remove outliers and to ensure that language markers identified in our analysis generalize to out-of-sample instances^43,44^.

#### Open-vocabulary

We used the MALLET implementation of latent Dirichlet allocation (LDA)^45^ to identify latent data-driven word clusters (topics). LDA has been found to offer superior predictive power relative to closed-vocabulary methods like General Inquirer (GI) and Linguistic Inquiry and Word Count (LIWC)^46^. The topics are open-source^16^ and are generated on a corpus of about 18 million Facebook posts with alpha adjusted to 0.30 to favor fewer topics per document. We represent each user in terms of their probability of mentioning each of the 2000 topics as (p(topic, user), which is derived from their probability of mentioning a word(p(word|user)) and the probability of the words being in given topics (p(topic|word)).

Inherently, each topic is realized as a set of words with probabilities. Every individual is thus scored based on their likelihood of mentioning each of the 2000 topics (p(topic, user), which is derived from the probability of the individual mentioning a word (p(word|user)) and the probability of the word appearing in a given topic (p(topic|word)).

#### Linguistic inquiry and word count (LIWC)

For robustness, we also compared our results from LDA with the 2022 version of LIWC (LIWC-22), a closed-vocabulary method^47^. LIWC offers similar extraction of linguistic features as MALLET but instead consists of 102 theory-based, manually-curated categories, and is one of the most popular closed-vocabulary methods within psychology^46,47^. Because our focus is on the open-vocabulary approach, we prioritize discussing and interpreting these results but do present LIWC corresponding findings as well.

### Statistical analysis

Each user’s language represented as a dimensional vector was used as the input, and the degree of work-to-personal or personal-to-work conflict was used as the output in an ordinary least squares regression. The degree of work-to-personal or personal-to-work conflict was computed as the mean score of the five questions of each subscale. We also added demographic variables (age, gender, education, and personal income) as covariates to control for their influence on users’ language. We utilized Benjamini–Hochberg^48^ p-correction to correct for multiple hypothesis testing (false discovery rate) and used *p* < 0.05 to indicate meaningful correlations.

### Predictive modeling

We evaluated the feasibility of predicting the work-to-personal conflict and personal-to-work conflict dimensions based on the social media language and demographic conditions. We used topics and demographics as features, which were treated as independent variables in a machine learning algorithm (ridge regression) to predict the dependent variable (i.e., work-to-personal and personal-to-work conflict). We used ridge regression to validate the model in a five-fold cross-validation setting to avoid overfitting^46,49^. In each case, hyperparameter selection was performed in a cross-validation setting. The prediction performances are reported as Pearson correlation and Mean Absolute Error for the outcomes.

### Data availability and transparency

The datasets generated during and/or analyzed during the current study are available from the corresponding author upon reasonable request. As this collected data was part of a broader collaboration, one part of the data has been used for another journal publication. However, there are no overlaps in the scales used in this manuscript and the other publication. The other publication examines loneliness and depression, whereas we examine work-personal conflict here. We detail this in Appendix D.

## Results

### Predictive accuracy

We found that personal-to-work conflict (*r* = 0.23) was more predictable from Facebook posts than work-to-personal conflict (*r* = 0.15) when using topics. Combining topics with age, gender, education, and personal income yielded slightly higher predictions for personal-to-work conflict (Table 1).

**Table 1:** Predictability of personal-to-work and work-to-personal conflict.

	Personal-to-work mean	Work-to-personal mean
Topics only	0.23	0.15
Age + gender only	0.21	0.15
All demographics	0.20	0.16
Topics + all demographics	0.24	0.14

All demographics includes age, gender, education, and personal income.

### Linguistic analysis

In addition to predictability, we investigated themes that emerged for predicting work-personal conflict by running correlations between the 2000 topics, work-to-personal subscale score means, and personal-to-work subscale score means, controlling for age, gender, education, and personal income. We defined a theme as seven or more significant (*p* < 0.05) topics that were similar in content. There were three themes for predicting high personal-to-work conflict, two themes for predicting low personal-to-work conflict, and one theme for predicting low work-to-personal conflict. There were no themes for predicting high work-to-personal conflict. We compared these themes to the significant LIWC categories for predicting work-personal conflict. See Appendix B for all significant LIWC categories. We also compared vocabulary sizes between the high and low conflict individuals (Appendix C).

#### High personal-to-work conflict topics

Netspeak (“u,” “lol,” “wen,” “coz”), swearing (“shit,” “fuck,” “bitch,” “asshole”), and troubled language (“pain,” “losing,” “sanity,” “bothered”) predicted high personal-to-work conflict. For the netspeak theme, we included topics where at least three of the fifteen words in the topic were netspeak; there were 37 netspeak topics. For the swearing theme, we included topics that had at least three swear words; there were 9 such topics. For troubled language, we included topics related to pain and loss; there were 7 such topics. There was one topic (Topic 1573) that qualified as both a netspeak and a swearing topic, and one topic (Topic 1353) that qualified as both a netspeak and a troubled language topic. See Table 2 for illustrative topics for each theme. For LIWC, we found that the Netspeak and Swearing categories were also significant for predicting high personal-to-work conflict. LIWC does not have an analogous category to troubled language.

**Table 2:** Illustrative high personal-to-work conflict topics.

Theme	Illustrative topics	Illustrative posts	Effect size (95% CI)
Netspeak	ur, dont, cuz, wen, wat, ppl, kno, urself, wats, urs, isnt, tht, bc, rite, tru	“When ur up ur up n when ur down ur down ur down :/”	0.10 (0.06, 0.13)
	bt, dat, ur, wen, nt, lyf, lyk, luv, dnt, dis, cn, wid, sum, dey, hav	“Lmmfao right nt all bt most”	0.09 (0.06, 0.13)
	ur, urself, u'll, coz, u've, cos, urs, bcoz, wht, givin	“U've been warned!”	0.09 (0.05, 0.12)
Swearing and derogatory language	shit, fuck, fucked, fucking, damn, ass, fuckin, bullshit, bitch, shitty, pissed, shits, dude, bitches, fucks	“who's fucked….im fucked”	0.10 (0.07, 0.14)
	shit, damn, ass, fuk, hell, wtf, fuck, dam, fuckin, bitch, pissed, aint, hella, dumb, piss	“What in the actual fuk”	0.10 (0.06, 0.14)
	shit, ass, bitches, fuck, bitch, niggas, nigga, hoes, aint, yo, hoe, dumb, fake, fuckin, yall	“hoes a be hoes. #morals”	0.09 (0.05, 0.13)
Troubled	mind, lose, losing, lost, control, sight, loose, loosing, sanity, grip, temper, ability, wanting, interest, slowly	“is losing ittt”	0.10 (0.06, 0.13)
	dont, wanna, talk, leave, anymore, cuz, mess, bothered, cus, ughh, ='(	“Dont wanna leave”	0.09 (0.05, 0.12)
	inside, deep, feel, heart, pain, hide, eyes, soul, empty, broken, pride, lies, trapped, skin, lie	“Trapped Inside My Head”	0.08 (0.04, 0.11)

Topics were categorized into themes based on qualification criteria. These illustrative topics are significant (*p* < 0.05) after Benjamini–Hochberg p-correction. The complete list of significant topics can be found in Appendix A.

Notably, there were no low personal-to-work conflict topics with netspeak and swearing, suggesting that netspeak and swearing are reliable indicators of high personal-to-work conflict.

#### Low personal-to-work conflict topics

Language about work (“work,” “job,” “overtime,” “paycheck”) and positivity (“amazing,” “fantastic,” “excited,” “woohoo”) predicted low personal-to-work conflict. For the work theme, we included topics that contained the word “work” or “job”; there were 13 work topics. The positivity theme included both topics with positive adjectives (“amazing,” “fantastic,” “wonderful”) and words conveying excitement (“excited,” “woohoo,” “yay”). Importantly, we did not count topics that had any negative words even if they were largely positive otherwise. For example, Topic 522, which includes both “excited” and “dreading,” and Topic 793, which includes both “yay” and “bleh,” were not considered positivity topics. There were 14 positivity topics. There were three topics (Topics 420, 1831, and 1862) that qualified as both a work topic and a positivity topic. See Table 3 for illustrative topics for each theme. For LIWC, we found that the Work category was not significant for predicting low work-to-personal conflict. Positive Emotion and Positive Tone, the two categories analogous to our positivity theme, were also not significant for predicting low work-to-personal conflict.

**Table 3:** Illustrative low personal-to-work conflict topics.

Theme	Illustrative topics	Illustrative posts	Effect size (95% CI)
Work	Today, work, tomorrow, lunch, yay, meeting, noon, productive, payday, picking, client	“Meeting then lunch”	− 0.09 (− 0.05, − 0.13)
	Work, hours, working, week, worked, overtime, paycheck, paid, extra, schedule, exhausted, atleast, ot, shifts, volunteer	“is working overtime”	− 0.08 (− 0.05, − 0.12)
Positivity	Amazing, fantastic, absolutely, fabulous, brilliant, gorgeous, towers, excellent, spectacular, simply, amazingly, absolutly, incredible, wonderfully, amazin	“Brilliant”	− 0.09 (− 0.06, − 0.13)
	Amazing, boyfriend, wonderful, absolutely, girlfriend, simply, lucky, incredible, gorgeous, fiance, fantastic, amazingly, amazin, incredibly, wonderfully	“Absolutely amazing”	− 0.08 (− 0.05, − 0.12)
	Night, amazing, awesome, pretty, epic, wow, fantastic, gig, incredible, ended, fireworks, twas, brilliant, spectacular, wicked	“Awesome”	− 0.08 (− 0.04, − 0.11)

Topics were categorized into themes based on qualification criteria. These illustrative topics are significant (*p* < 0.05) after Benjamini–Hochberg p-correction. The complete list of significant topics can be found in Appendix A.

#### High work-to-personal conflict topics

There were two significant (*p* < 0.05) high work-to-personal conflict topics. The first topic included the following words: “feels,” “weird,” “kinda,” “feel,” “feeling,” “wierd,” “bit,” “felt,” “strange,” “odd,” “hmm,” “suddenly,” “sort,” “awkward,” “dunno.” The second topic included the following words: “kinda,” “sad,” “sorta,” “sucks,” “bummed,” “feelin,” “sucked,” “lame,” “upset,” “depressing,” “pissed,” “tho,” “scary,” “disappointed,” “depressed.” Because we defined a theme as *seven or more* topics that were similar in content, we did not count this as a theme. However, when conducting LIWC, we did find two categories—Negative Emotion and Negative Tone—that were similar to these two topics and were significant for predicting high work-to-personal conflict.

#### Low work-to-personal conflict topics

Language about birthdays (“birthday,” “wishes,” “happy,” “celebrate”) emerged as the single theme for predicting low work-to-personal conflict. See Table 4 for illustrative topics for the birthday theme. LIWC does not have an analogous category to birthdays. For LIWC, the Positive Emotion category was significant for predicting low work-to-personal conflict.

**Table 4:** Illustrative low work-to-personal conflict topics.

Theme	Illustrative topics	Illustrative posts	Effect size (95% CI)
Birthdays	Happy, birthday, wishing, sister, years, wonderful, st, daughter, nephew, brother, son, turns, niece, special, celebrate	“Happy Birthday Brother”	− 0.09 (− 0.05, − 0.12)
	Party, birthday, bday, b-day, surprise, celebrate, st, saturday, celebrating, bash, cake, blast, celebration, graduation, planning	“Party party party(:”	− 0.08 (− 0.04, − 0.12)
	Birthday, happy, wishes, b-day, celebrate, celebrating, birthdays, present, celebration, cake, celebrated, mom's, belated, dad's, brother's	“Celebrating Marge's B-Day!”	− 0.08 (− 0.04, − 0.12)
	Birthday, wishes, happy, present, wished, cheers, birthdays, celebration, belated	“Thanks for the birthday wishes”	− 0.07 (− 0.04, − 0.11)

Topics were categorized into themes based on qualification criteria. These illustrative topics are significant (*p* < 0.05) after Benjamini–Hochberg p-correction. The complete list of significant topics can be found in Appendix A.

## Discussion

In this study, we built a language model that can predict work-personal conflict levels and uncovered important insights about work-personal conflict using Facebook status posts. The level of prediction (*r* = 0.15–0.23) is similar to past research examining the use of social media language in predicting individual difference constructs, such as personality (*r* = 0.21–0.42), life satisfaction (*r* = 0.14–0.19), and stress (*r* = 0.12–0.21). The ability to predict work-personal conflict is pivotal, as tracking work-personal conflict through social media enables organizations and communities to understand their workers’ collective experiences over time and contribute to broader well-being and economic indices.

In terms of which component was more predictable from social media language, we found that personal-to-work conflict was more predictable than work-to-personal conflict. The higher predictability of personal-to-work conflict was interesting, though not entirely surprising, given that personal-to-work conflict is related to satisfaction with the need for relatedness^9^, a primary reason cited for social media use, while work-to-personal conflict is not. Additionally, Facebook is considered to be a “personal” social media platform where connections and posts shared are more centered around an individual’s personal life compared to more work-focused platforms (e.g., LinkedIn)^50,51^.

Critically, the use of social media language analysis enabled deeper insights into the phenomenology of work-personal conflict as we could identify key topics (via LDA) and categories (via LIWC) that are discussed online by individuals. We found that high personal-to-work and high work-to-personal conflict were associated with negative language—swearing and troubled language topic themes for the former, and Negative Emotion and Negative Tone LIWC categories for the latter. On the other hand, we found that low personal-to-work and low work-to-personal conflict were associated with positive language—the positivity topic theme for the former, and the Positive Emotion LIWC category for the latter.

The relationship between negative language and high conflict—and between positive language and low conflict—is in line with past research on emotions and conflict. Previous studies have found that negative emotions are associated with work-family conflict, lower job satisfaction, lower marital satisfaction, and higher stress^52–54^. Positive emotions, on the other hand, are associated with better work relationships, better work outcomes, and lower stress^55–57^.

Work topics as an indicator of low personal-to-work conflict may be explained by life satisfaction research. Studies show that individuals are generally only able to focus on some (not all) domains, which leads to deprioritizing the other domains and varying degrees of satisfaction between different domains^58,59^. It is possible that individuals who post about work invest a lot of energy in that domain, leading to less focus on the personal domain and, subsequently, less conflict from the personal domain to other domains. Although the Work LIWC category was not significant (*p* = 0.15) for predicting low personal-to-work conflict, there was strong evidence from the open-vocabulary approach, which was the focus of our study.

Additionally, birthdays as the single topic theme for low work-to-personal conflict may be explained with similar logic. One explanation for the birthday theme is that birthdays are considered personal and family-oriented and not work-oriented. Given that individuals are generally only able to focus on some domains while de-prioritizing others^58,59^, it is possible that individuals who post about birthdays do not invest much in the work domain and therefore have less conflict from the work domain to other domains.

The birthday topic theme finding is an example of how using organic data can reveal nuanced individual differences and provide details about people’s daily interactions in ways that most other methodologies cannot, as the relationship between birthday posts and work-personal conflict would be difficult to glean from traditional surveys or lab experiments. The netspeak topic theme finding is another example; the relationship between netspeak and work-personal conflict would be difficult to glean from other methods.

### Future directions

Future research could investigate the relationship between social media language use, work-personal conflict, and other measures, such as perceived stress or job satisfaction. Researchers could also compare work-personal conflict in language use across different languages and locales, as this study focused on English posts from the United States. Additionally, the study of work-personal conflict and language use could be expanded to other social media platforms. Given that we are investigating the work domain, platforms like Reddit, Glassdoor, and LinkedIn could be particularly insightful.

Another potential extension of our research could be investigating work-personal conflict and language use following certain events, interventions, or policy implementations. During the COVID-19 pandemic, there were several studies conducted that investigated the effect of mandatory remote work policies on work-life balance^60,61^ and social media sentiment^22,25,62^. It could be interesting to evaluate the impact of remote work on work-personal conflict levels and social media language in situations where individuals voluntarily choose to work remotely compared to situations in which individuals are mandated to do so.

### Limitations

Our study was limited in several ways. Because of the small number of individuals (*n* = 11) who identified outside of the gender binary (i.e., did not identify as a man or a woman), we removed them from our analytic sample as we would not have been able to compute statistically significant gender differences for the non-binary group. We also removed non-English posts and users who did not have at least 500 words across their Facebook status posts in English. This decision was made in order to avoid the confounds of bilingualism. The study was also limited to the subset of the population that has a Facebook account, which may differ from the general population in significant ways (e.g., political beliefs)^63–65^.

Additionally, Facebook status posts may not tell the complete story of an individual’s work-personal conflict. While posts provide a glimpse into an individual’s activities, moods, and thoughts, it is limited to those that the individual is willing to share publicly or with their broader network^13,65^. There is research showing that workers are becoming increasingly worried about current and prospective employers reading their social media posts and are consequently sharing less or different content on social media with that possibility in mind^66–69^. Despite this, it is fascinating that work-personal conflict can still be predicted by language in these posts.

## Conclusion

As communication technology continues to advance and more organizations move toward remote work and similar policies, the line between work and personal life may become even blurrier, bringing work-personal conflict issues to the forefront. In this article, we provided evidence that social media language data can be used to predict and track work-personal conflict in a novel and non-intrusive way. Given the connection between work-personal conflict and general well-being, this area of research will remain critical in psychology in the years to come.

### Supplementary Information

Supplementary Tables.
