# Supplementary material for: The online language of work-personal conflict
Source: Sci Rep. 2023 Nov 29;13:21019. doi: 10.1038/s41598-023-48193-3 (PMC10686985; doi:10.1038/s41598-023-48193-3)
Supplement: Supplementary file 1 — Supplementary Tables. [file 41598_2023_48193_MOESM1_ESM.docx]

**Appendix A**

**All Significant Conflict Topics**

**Table A1**

*All Significant High Personal-to-Work Conflict Topics*

| **Top Words** | ***p*-value** | **Effect Size** | **95% CI (lower)** | **95% CI (upper)** |
| --- | --- | --- | --- | --- |
| wah, yeh, ah, hai, wa, ke, le, mai, wel, ye, eh, mere, chain, rang, bo | 0.0004 | 0.10 | 0.06 | 0.13 |
| ur, dont, cuz, wen, wat, ppl, kno, urself, wats, urs, isnt, tht, bc, rite, tru | 0.0005 | 0.09 | 0.06 | 0.13 |
| lol, nah, haha, hahaha, kidding, hahah, hah, lmao, yah, eh, hahahaha, hmm, jk, hmmm, naw | 0.0006 | 0.10 | 0.06 | 0.13 |
| eid, mubarak, egypt, al, grand, auto, wishing, allah, revolution, jordan, muslim, advance, miserable, muslims, peaceful | 0.0012 | 0.09 | 0.05 | 0.12 |
| bt, dat, ur, wen, nt, lyf, lyk, luv, dnt, dis, cn, wid, sum, dey, hav | 0.0012 | 0.09 | 0.05 | 0.12 |
| dont, somthing, belive, ill, wont, realy, ther, didnt, ive, untill, iv, ment, isnt, happend, fucked | 0.0012 | 0.09 | 0.05 | 0.12 |
| ur, urself, u'll, coz, u've, cos, urs, bcoz, wht, givin | 0.0012 | 0.08 | 0.05 | 0.12 |
| die, ek, en, wat, te, om, net, van, kan, op, se, met, al, dis, wil | 0.0012 | 0.08 | 0.05 | 0.12 |
| ur, wen, hav, dat, wat, wud, coz, dis, wid, cud, wil, luv, bcoz, sum, ppl | 0.0016 | 0.08 | 0.04 | 0.12 |
| lol, im, lo, omg, wow, yea, dork, hyper, lmao, couldnt, addicted, nerd, hype, offically, kidding | 0.0017 | 0.09 | 0.05 | 0.12 |
| live, die, life, forever, you'll, dream, lived, notice, born, learn, fullest, fight, dreams, potential, smiling | 0.0017 | 0.08 | 0.04 | 0.12 |
| stay, leave, lives, forever, tuned, focused, awhile, strong, footprints, hearts, touch, quickly, fade, classy, searching | 0.0019 | 0.08 | 0.04 | 0.12 |
| im, ill, dont, ive, cuz, iv, id, havent, youll, couldnt, alot, whos, isnt, itll, coz | 0.0019 | 0.08 | 0.05 | 0.12 |
| shit, fuck, fucked, fucking, damn, ass, fuckin, bullshit, bitch, shitty, pissed, shits, dude, bitches, fucks | 0.0020 | 0.08 | 0.05 | 0.12 |
| el, ya, ma, ana, wa, al, ba, ala, mn, ad, bs, ta, er, eh, ra | 0.0025 | 0.08 | 0.04 | 0.11 |
| da, dat, wit, dis, dey, aint, jus, cuz, bout, wat, kno, nigga, dnt, sum, dem | 0.0025 | 0.08 | 0.04 | 0.12 |
| yall, bout, cuz, ill, ima, aint, kno, lmao, imma, haters, holla, smh, dang, folks, talkin | 0.0025 | 0.08 | 0.04 | 0.12 |
| dont, wanna, talk, leave, anymore, cuz, mess, bothered, cus, ughh, ='( | 0.0026 | 0.08 | 0.05 | 0.12 |
| shit, holy, fuck, fucking, piece, bull, load, fuckin, ton, outta, pile, worthless, goddamn, scares, stink | 0.0026 | 0.08 | 0.04 | 0.12 |
| hai, ki, se, ko, ka, ho, ke, nahi, bhi, mein, dil, main, hain, kya, jo | 0.0026 | 0.08 | 0.04 | 0.11 |
| im, gona, wana, dont, realy, cus, gota, coz, tomorow, whos, ther, outa, tonite, finaly, tis | 0.0026 | 0.08 | 0.04 | 0.11 |
| respect, give, show, brought, country, england, earned, minutes, fight, deserve, boys, union, group, lives, showing | 0.0026 | 0.07 | 0.04 | 0.11 |
| shit, damn, ass, fuk, hell, wtf, fuck, dam, fuckin, bitch, pissed, aint, hella, dumb, piss | 0.0027 | 0.08 | 0.04 | 0.12 |
| mind, lose, losing, lost, control, sight, loose, loosing, sanity, grip, temper, ability, wanting, interest, slowly | 0.0027 | 0.08 | 0.04 | 0.11 |
| bitch, fuck, karma, ass, slap, son, lifes, life's, dumb, hoe, pimp, sexy, bitches, slapped, slut | 0.0028 | 0.08 | 0.04 | 0.11 |
| application, job, college, applications, filling, fill, form, online, paperwork, filled, forms, finished, process, scholarship, apply | 0.0028 | 0.07 | 0.04 | 0.11 |
| xa, d: | 0.0028 | 0.07 | 0.04 | 0.11 |
| fucking, fuck, shit, pissed, bullshit, goddamn, piss, bitch, wtf, asshole, fucked, >:, pissing, shitty, pisses | 0.0030 | 0.08 | 0.04 | 0.11 |
| nigga, aint, ya, wit, niggas, yo, gucci, bitch, shawty, tha, yall, gon, tryna, em, ain't | 0.0030 | 0.08 | 0.04 | 0.11 |
| lol, lo, tho, fun, gotta, damn, guess, gots, messing, welp, dang, prob, couldnt, tooo, awwww | 0.0031 | 0.08 | 0.04 | 0.11 |
| nyt, nite, wot, gd, gud, xx, tho, sum, bk, shud, xxx, cnt, wif, wi, iv | 0.0031 | 0.07 | 0.04 | 0.11 |
| allah, peace, muslim, wa, al, deeds, paradise, reward, messenger, beloved, sins, blessings, prayer, mercy, grant | 0.0031 | 0.07 | 0.04 | 0.11 |
| shit, ass, bitches, fuck, bitch, niggas, nigga, hoes, aint, yo, hoe, dumb, fake, fuckin, yall | 0.0032 | 0.08 | 0.04 | 0.11 |
| lol, tho, cool, funny, lo, cuz, kinda, idk, lmao, hella, mad, haha, crazy, smh, chill | 0.0036 | 0.08 | 0.04 | 0.11 |
| life, live, enjoy, moment, short, fullest, regrets, everyday, living, chances, cherish, regret, life's, granted, avoid | 0.0036 | 0.07 | 0.03 | 0.11 |
| wont, dont, ill, give, leave, till, anymore, promise, untill, prob, prolly, atleast, bother, bet, anytime | 0.0036 | 0.08 | 0.04 | 0.11 |
| fuck, shit, fucking, shut, sake, bitches, bullshit, outta, fucks, haters, fuckers, outa | 0.0042 | 0.07 | 0.04 | 0.11 |
| mi, dem, ah, fi, di, jus, nah, dis, wid, dat, ting, meh, wah, kno, wi | 0.0048 | 0.07 | 0.03 | 0.11 |
| wid, gud, ma, tym, frnds, fr, dis, luv, frm, lyf, bt, thnx, hav, hv, nw | 0.0052 | 0.07 | 0.03 | 0.11 |
| hearts, birth, kingdom, gave, souls, minds, giving, bodies, certificate, control, beat, death, heads, remembered, gods | 0.0053 | 0.07 | 0.03 | 0.11 |
| ill, im, guess, fine, idk, admit, itll, mayb, settle, untill, tomorow, idc | 0.0055 | 0.07 | 0.03 | 0.11 |
| imma, im, ima, cuz, gon, yea, bout, idk, aint, swear, lovin, outta, beast, lookin, shawty | 0.0057 | 0.07 | 0.04 | 0.11 |
| na, mo, ka, ko, ang, sa, ako, lang, kung, ng, hindi, di, pa, mahal, kita | 0.0060 | 0.07 | 0.03 | 0.11 |
| dream, reality, dreams, wake, dreaming, real, true, nightmare, vision, living, imagination, waking, fantasy, realize, thoughts | 0.0064 | 0.07 | 0.03 | 0.11 |
| guys, pls, hey, thx, ur, asap, btw, thnx, reply, suggest, inform, esp, peeps, msg, contact | 0.0065 | 0.07 | 0.03 | 0.10 |
| live, lives, dies, pooh, living, happily, longer, harmony, minus, lived, hundred, poo, stream, lifestyle, saves | 0.0066 | 0.07 | 0.03 | 0.10 |
| inside, deep, feel, heart, pain, hide, eyes, soul, empty, broken, pride, lies, trapped, skin, lie | 0.0067 | 0.07 | 0.03 | 0.10 |
| fucking, fuck, shit, hate, stupid, retarded, bitch, pissed, omfg, kidding, shitty, bitches, annoying, bullshit, idiot | 0.0071 | 0.07 | 0.03 | 0.11 |
| da, mi, wit, dnt, meh, bout, sum, tha, bt, dis, dat, wat, iz, wen, ta | 0.0075 | 0.07 | 0.03 | 0.11 |
| live, love, life, laugh, learn, breathe, simply, motto, deeply, continue, regret, kindly, intention, edge, fully | 0.0079 | 0.07 | 0.03 | 0.10 |
| ya, wanna, holla, gotta, yo, boi, hit, lemme, hey, ill, ya'll, peeps, somethin, kno, gurl | 0.0080 | 0.07 | 0.03 | 0.11 |
| mark, points, marks, question, bonus, twain, stretch, brownie, period, spot, repeat, halfway, brilliant, earned, pause | 0.0084 | 0.07 | 0.03 | 0.10 |
| hug, kiss, give, chance, relationship, cuddle, laugh, chill, friendship, apology, hugs, inbox, sex, cheating, term | 0.0086 | 0.07 | 0.03 | 0.10 |
| ppl, dont, hate, talk, cuz, smh, alot, act, dumb, sm, piss, wen, ppls, fake, sayin | 0.0087 | 0.07 | 0.03 | 0.10 |
| lol, jk, lmao, idk, funn, lmfao, lolz, kidding, ily, hahahahaha, sorta, rofl, ewww, kool, hahahahahaha | 0.0088 | 0.07 | 0.03 | 0.10 |
| na, ko, ng, ako, sa, ang, pa, lang, naman, di, wala, sana, hindi, bukas, kaya | 0.0088 | 0.07 | 0.03 | 0.10 |
| na, ka, mo, ko, ako, ba, ang, sa, si, naman, pa, lang, ng, nga, yan | 0.0088 | 0.07 | 0.03 | 0.10 |
| im, tired, sick, ugh, soo, bein, bs, bullshit, hearing, stressed, tire, dealing, lied, ppls, grrrrrrr | 0.0091 | 0.07 | 0.03 | 0.11 |
| nu, ne, te, di, aa, tu, main, ki, ni, dil, vi, ch, ke, meri, gal | 0.0095 | 0.07 | 0.03 | 0.10 |
| sa, na, ng, ko, mga, po, ako, lahat, ang, salamat, nyo, lang, kayo, pa, ung | 0.0097 | 0.06 | 0.03 | 0.10 |
| stand, can't, fall, i'll, tall, sit, standing, strong, respond, saving, awe, stands, crowd, sends, firm | 0.0105 | 0.06 | 0.03 | 0.10 |
| ang, sa, ng, na, mga, mo, ay, hindi, ko, ka, lang, kung, lahat, para, pa | 0.0106 | 0.06 | 0.03 | 0.10 |
| im, bore, bored, soo, sooo, hella, tire, soooo, soooooo, sooooooo, sooooooooo, atm, effin, uber, sooooooooooo | 0.0119 | 0.06 | 0.03 | 0.10 |
| na, sa, din, ang, ko, tapos, lang, naman, ako, rin, hahaha, yey, sana, bukas, hay | 0.0119 | 0.06 | 0.03 | 0.10 |
| na, ki, ta, er, ar, aj, din, mon, hoy, sob, jay, je, por, te, ai | 0.0119 | 0.06 | 0.03 | 0.10 |
| real, quick, easy, question, shady, slim, fast, hurry, here's, poll, honest, healthy, recovery, survey, snap | 0.0119 | 0.06 | 0.03 | 0.10 |
| dollar, dollars, million, bucks, worth, penny, money, cents, cost, buy, spent, hundred, pound, bill, ten | 0.0132 | 0.06 | 0.03 | 0.10 |
| kiss, touch, lips, eyes, hold, hand, kissed, mine, taste, soft, cheek, warm, breath, kissing, skin | 0.0136 | 0.06 | 0.03 | 0.10 |
| jesus, died, christ, cross, thinking, repost, let's, ashamed, lift, prove, statement, challenge, savior, stand, gospel | 0.0138 | 0.06 | 0.03 | 0.10 |
| lol, lo, fun, hah, atleast, btw, soooo, oops, remeber, gosh, hahahah, xp, horn, sooooo, ahh | 0.0139 | 0.06 | 0.03 | 0.10 |
| im, tired, glad, ready, soooo, liking, alittle, exited, greatful, obsessed, beleive, sucky | 0.0143 | 0.07 | 0.03 | 0.10 |
| im, goin, tired, rite, pissed, feelin, idk, gettin, confused, wont, tho, dam, hype, sleepin, diff | 0.0144 | 0.07 | 0.03 | 0.10 |
| dont, worry, whats, coz, beleive, wan, atleast, grrr, arent, tooo, piss, hasnt, hows, ummmm, shouldnt | 0.0144 | 0.06 | 0.03 | 0.10 |
| mo, te, ar, ne, ti, au, pa, ou, ta, ki, li, se, le, pu, po | 0.0144 | 0.06 | 0.03 | 0.10 |
| die, live, born, cry, kill, young, i'd, ways, fluffy, lie, alive, original, dies, survive, boredom | 0.0149 | 0.06 | 0.03 | 0.10 |
| da, nw, gt, wrk, bt, jst, cnt, nt, bk, frm, jus, dis, nxt, wk, gud | 0.0151 | 0.06 | 0.02 | 0.10 |
| de, te, el, la, mi, en, tu, es, se, si, por, yo, amor, una, con | 0.0168 | 0.06 | 0.02 | 0.10 |
| im, dont, anymore, idk, confused, whats, wont, isnt, alot, honestly, idc, mad, ive, upset, arent | 0.0176 | 0.07 | 0.03 | 0.10 |
| ja, iv, nyt, ss, pit, ty, ole, ku, tt, vain, vi, kin, mist, eli, ist | 0.0203 | 0.06 | 0.02 | 0.10 |
| lol, lo, haha, didnt, soo, dang, ahhh, forgot, hmm, geez, ahhhh, lastnight, gosh, =/, fyi | 0.0210 | 0.06 | 0.03 | 0.10 |
| wanna, dont, join, hang, chill, kinda, somethin, badly, dun, lemme, takers, hangout, lend, skip, jogging | 0.0212 | 0.06 | 0.02 | 0.10 |
| tooo, sooo, soooo, alll, sooooo, toooo, goood, meee, meeee, youuu, gooo, soooooo, allll, gooood, ohhh | 0.0212 | 0.06 | 0.02 | 0.10 |
| de, si, sa, nu, la, cu, ca, pe, mai, ce, se, din, fi, va, dar | 0.0220 | 0.06 | 0.02 | 0.10 |
| wanna, don't, stay, touch, hear, lonely, babe, undo, blew, stole, explode, surrender, stare, brag, consuming | 0.0264 | 0.06 | 0.03 | 0.10 |
| plz, ur, ppl, hey, msg, pray, comment, txt, reply, ans, sum, pls, asap, coz, thx | 0.0265 | 0.06 | 0.02 | 0.09 |
| sa, na, ko, ang, ka, pa, nga, mga, ni, lang, kay, ta, akong, wala, lng | 0.0265 | 0.06 | 0.02 | 0.09 |
| pain, hurt, pleasure, meds, killers, ease, gain, caused, hurts, pains, worse, chest, pills, numb, hurting | 0.0265 | 0.06 | 0.02 | 0.09 |
| wa, ni, wo, ga, ai, mo, yo, ne, japanese, ima, shite, demo, ama, baka, mata | 0.0276 | 0.06 | 0.02 | 0.09 |
| de, um, para, em, se, os, mas, por, dia, ser, est, ou, como, dos, vida | 0.0289 | 0.06 | 0.02 | 0.09 |
| ng, ang, ko, sa, sakit, sarap, ulo, pa, saya, hirap, naman, grabe, hay, talaga, mag | 0.0290 | 0.06 | 0.02 | 0.09 |
| fuck, fucking, shit, bitch, ass, fuckin, asshole, cunt, dick, pussy, bitches, motherfucker, assholes, piss, dumb | 0.0300 | 0.06 | 0.02 | 0.10 |
| im, bored, tired, sooo, hungry, soooo, sleepy, sooooo, ugh, soooooo, bore, hyper, tire, sooooooooo, extremely | 0.0303 | 0.06 | 0.02 | 0.09 |
| money, buy, spend, save, spending, spent, happiness, earn, saving, cash, pocket, rich, clothes, cars, makin | 0.0303 | 0.06 | 0.02 | 0.09 |
| im, tired, soo, sooo, bored, tire, soooo, freakin, ugh, ughh, ughhh, stressed, sooooo, bore, effin | 0.0307 | 0.06 | 0.02 | 0.10 |
| yo, miss, momma, mama, fat, tube, homie, mamma, homies, aye, jokes, soy, lemme, hahahahahahaha, gangsta | 0.0307 | 0.06 | 0.02 | 0.09 |
| tears, eyes, cry, tear, smile, pain, shed, crying, cried, wipe, dry, fears, hide, smiles, brought | 0.0338 | 0.06 | 0.02 | 0.09 |
| ya, dig, betta, tellin, buddy, yer, comin, hells, sista, shoulda, spit | 0.0351 | 0.06 | 0.02 | 0.09 |
| de, la, el, en, es, los, se, para, por, las, con, una, del, mi, si | 0.0357 | 0.06 | 0.02 | 0.09 |
| dead, bin, alive, walking, drop, laden, osama, killed, living, bodies, buried, gorgeous, obama, rising, fred | 0.0369 | 0.06 | 0.02 | 0.09 |
| bored, im, txt, text, talk, meh, hmu, bore, kinda, somethin, fone, soooo, entertain, plz, hm | 0.0369 | 0.06 | 0.02 | 0.09 |
| alive, survive, live, moment, dead, die, longer, keeping, buried, prize, sexiest, survived, disappear, humans, bleed | 0.0390 | 0.06 | 0.02 | 0.09 |
| funny, lol, haha, lmao, funn, hahaha, omg, sooo, soo, hilarious, watchin, lmfao, hahah, hella, hah | 0.0416 | 0.06 | 0.02 | 0.09 |
| im, glad, dont, ive, scared, didnt, tho, guessing, wasnt, couldnt, shouldnt, unsure, grounded, cus, terrified | 0.0425 | 0.06 | 0.02 | 0.09 |
| aint, nothin, shit, cuz, doin, gotta, somethin, ain't, gon, bout, trippin, bitch, workin, thang, gettin | 0.0434 | 0.06 | 0.02 | 0.09 |
| ng, lng, na, ang, sa, aq, ko, ung, mga, nmn, pa, nman, tlga, mu, kc | 0.0436 | 0.05 | 0.02 | 0.09 |
| de, je, le, la, est, les, pas, pour, en, ne, vous, des, mon, il, ce | 0.0446 | 0.05 | 0.02 | 0.09 |
| ta, ma, li, il, min, te, bo, pa, cu, imma, mal, su, nan, tin, den | 0.0449 | 0.05 | 0.02 | 0.09 |
| dont, care, understand, anymore, worry, bother, talk, doesnt, kno, judge, expect, act, honestly, wana, pretend | 0.0466 | 0.06 | 0.02 | 0.09 |
| da, wit, bout, jus, ta, dis, sum, nite, sn, lil, ma, goin, gud, dat, wat | 0.0468 | 0.05 | 0.02 | 0.09 |
| extra, soldier, credit, class, months, enjoys, everyday, flies, country, sees, promise, support, reduced, defend, justify | 0.0473 | 0.05 | 0.02 | 0.09 |
| mum, dad, mummy, xx, mums, nan, xxx, cos, dads, loads, mu, whilst, realised, xxxx, dad's | 0.0488 | 0.05 | 0.02 | 0.09 |
| im, gonna, dont, ive, swear, havent, doesnt, tellin, joking | 0.0490 | 0.06 | 0.02 | 0.09 |

**Table A2**

*All Significant Low Personal-to-Work Conflict Topics*

| **Top Words** | ***p*-value** | **Effect Size** | **95% CI (lower)** | **95% CI (upper)** |
| --- | --- | --- | --- | --- |
| yay, excited, finally, woohoo, cap, graduation, coming, weeks, braces, dressing, picked, excitement, ordered, exciting, rehearsal | 0.0012 | -0.09 | -0.05 | -0.13 |
| school, tomorrow, closed, schools, snow, kids, cancelled, district, cancel, canceled, county, delay, tomorow, school's, due | 0.0012 | -0.09 | -0.05 | -0.12 |
| work, hours, working, week, worked, overtime, paycheck, paid, extra, schedule, exhausted, atleast, ot, shifts, volunteer | 0.0012 | -0.08 | -0.05 | -0.12 |
| tomorrow, school, starts, excited, tuesday, dreading, starting, yipee, bummer, noooo | 0.0019 | -0.08 | -0.05 | -0.12 |
| officially, summer, started, yay, decided, begun, vacation, offically, moved, woohoo, official, begin, registered, certified, announce | 0.0019 | -0.08 | -0.05 | -0.12 |
| hours, couple, days, weeks, ago, minutes, months, spend, happened, move, pieces, situation, seconds, floor, could've | 0.0025 | -0.08 | -0.04 | -0.11 |
| tomorrow, school, start, ready, classes, excited, starting, orientation, summer, wednesday, college, tuesday, nervous, kindergarten, internship | 0.0025 | -0.08 | -0.05 | -0.12 |
| hours, minutes, days, counting, till, seconds, left, minus, mins, til, starts, ten, minute, countdown, untill | 0.0026 | -0.08 | -0.04 | -0.11 |
| school, starts, tomorrow, week, summer, monday, weeks, tuesday, wednesday, thursday, semester, tomorow, registration, kindergarten, yikes | 0.0026 | -0.08 | -0.04 | -0.12 |
| dress, prom, shopping, wedding, dresses, wear, bought, fancy, shoes, formal, homecoming, outfit, rehearsal, clothes, dressed | 0.0026 | -0.08 | -0.04 | -0.11 |
| start, week, monday, job, starting, school, excited, starts, tuesday, training, classes, college, orientation, woohoo, january | 0.0027 | -0.08 | -0.04 | -0.11 |
| today, work, tomorrow, lunch, yay, meeting, noon, productive, payday, picking, client | 0.0031 | -0.07 | -0.04 | -0.11 |
| today, didn't, earlier, yesterday, couldn't, ended, planned, expected, usual, started, decided, wasn't, turned, played, showed | 0.0031 | -0.07 | -0.04 | -0.11 |
| school, high, middle, elementary, grade, reunion, graduate, schools, students, junior, teachers, jr, classmates, hs, graduated | 0.0033 | -0.07 | -0.04 | -0.11 |
| hour, minutes, ago, hours, half, minute, mins, left, min, drive, woke, ten, sit, late, started | 0.0036 | -0.07 | -0.04 | -0.11 |
| teacher, grade, class, student, teachers, teach, teaching, graders, preparing, parent, english, minute, students, future, mrs | 0.0045 | -0.07 | -0.04 | -0.11 |
| wouldn't, didn't, wasn't, weren't, knew, couldn't, wanted, i'd, hadn't, met, happened, crushed, surprised, explain, shouldn't | 0.0052 | -0.07 | -0.03 | -0.11 |
| hours, straight, sleep, days, couple, slept, worked, working, total, twelve, spent, exhausted, priorities, skip, fm | 0.0059 | -0.07 | -0.03 | -0.11 |
| class, period, mr, mrs, teacher, ms, homework, lunch, grade, english, math, spanish, science, french, pe | 0.0060 | -0.07 | -0.03 | -0.11 |
| months, years, days, ago, couple, weeks, haven't, past, started, havent, realized, eleven, twelve, dating, realised | 0.0065 | -0.07 | -0.03 | -0.10 |
| i've, years, changed, past, haven't, months, we've, times, found, you've, realized, hasn't, met, lived, moved | 0.0065 | -0.07 | -0.03 | -0.10 |
| work, night, tonight, working, shift, late, pm, closing, overnight, midnight, shifts, worked, picked, covering, thankfully | 0.0066 | -0.07 | -0.03 | -0.10 |
| tomorrow, school, day, forward, starting, meeting, exciting, hopes, dread, returning, yikes, grin, chin | 0.0067 | -0.07 | -0.03 | -0.11 |
| amazing, fantastic, absolutely, fabulous, brilliant, gorgeous, towers, excellent, spectacular, simply, amazingly, absolutly, incredible, wonderfully, amazin | 0.0072 | -0.07 | -0.03 | -0.11 |
| teachers, school, students, teaching, student, kids, teacher, parents, education, classroom, teach, college, exchange, schools, honor | 0.0084 | -0.07 | -0.03 | -0.11 |
| today, turned, realized, thought, started, yesterday, looked, didn't, felt, wasn't, woke, decided, happened, wanted, horrible | 0.0086 | -0.07 | -0.03 | -0.10 |
| great, lunch, nice, dinner, family, enjoyed, church, wonderful, afternoon, sunday, kids, evening, shopping, meeting, hubby | 0.0105 | -0.07 | -0.03 | -0.10 |
| camp, week, boot, summer, band, leaving, church, training, cheer, youth, volleyball, staff, pumped, cam, leadership | 0.0105 | -0.06 | -0.03 | -0.10 |
| wedding, married, royal, congrats, planning, reception, dress, congratulations, ring, bride, engagement, ceremony, cousin, engaged, maid | 0.0117 | -0.06 | -0.03 | -0.10 |
| great, job, guys, amazing, crew, awesome, show, cast, proud, congrats, fantastic, performance, excellent, rocked, congratulations | 0.0119 | -0.06 | -0.03 | -0.10 |
| night, amazing, awesome, pretty, epic, wow, fantastic, gig, incredible, ended, fireworks, twas, brilliant, spectacular, wicked | 0.0136 | -0.06 | -0.03 | -0.10 |
| back, work, tomorrow, school, ready, vacation, monday, weeks, dreading, sigh, yuck, routine, grind | 0.0143 | -0.06 | -0.03 | -0.10 |
| bell, taco, tacos, burrito, mexican, ring, ate, tuesday, nachos, saved, rings, craving, mmmm, margaritas, rang | 0.0144 | -0.06 | -0.02 | -0.10 |
| graduation, proud, congrats, congratulations, class, college, graduate, graduating, graduated, degree, officially, graduates, seniors, batch, grad | 0.0182 | -0.06 | -0.03 | -0.10 |
| camera, pictures, photo, photos, digital, taking, photography, picture, shoot, video, pics, film, cameras, photographer, professional | 0.0194 | -0.06 | -0.02 | -0.10 |
| amazing, boyfriend, wonderful, absolutely, girlfriend, simply, lucky, incredible, gorgeous, fiance, fantastic, amazingly, amazin, incredibly, wonderfully | 0.0210 | -0.06 | -0.02 | -0.10 |
| na, mo, ka, ko, ang, sa, ako, lang, kung, ng, hindi, di, pa, mahal, kita | 0.0212 | -0.06 | -0.02 | -0.10 |
| school, high, college, grad, musical, primary, middle, drama, career, graduated, graduation, elementary, school's, graduating, assembly | 0.0212 | -0.06 | -0.02 | -0.10 |
| box, chocolates, shiny, boxes, forest, names, empty, gum, cereal, opened, sharp, tissue, honesty, shaped, tool | 0.0212 | -0.06 | -0.02 | -0.10 |
| school, tomorrow, back, work, ready, yay, kiddos, yayyy | 0.0220 | -0.06 | -0.02 | -0.10 |
| nervous, excited, tomorrow, kinda, anxious, scared, starting, luck, breakdown, interview, wreck, audition, surgery, extremely, worried | 0.0228 | -0.06 | -0.03 | -0.10 |
| hour, shift, half, hr, delay, hours, shifts, worked, extra, hrs, twelve, workin, split, overtime, closing | 0.0263 | -0.06 | -0.02 | -0.09 |
| tomorrow, day, today, work, hoping, forward, it'll, dreading, technically | 0.0267 | -0.06 | -0.02 | -0.10 |
| work, till, til, pm, working, ish, midnight, workin, noon, close, 8:, visit, babysitting, 8p, worked | 0.0283 | -0.06 | -0.02 | -0.09 |
| hours, spent, half, hour, minutes, entire, past, wasted, couple, afternoon, literally, total, managed, er, countless | 0.0291 | -0.06 | -0.02 | -0.09 |
| notice, amy, suddenly, noticed, disappear, remembered, disappeared, realized, sign, magically, happened, they're, appears, wonders, looked | 0.0291 | -0.06 | -0.02 | -0.09 |
| should've, thought, didn't, could've, knew, wasn't, stayed, would've, shoulda, told, coulda, wanted, believed, woulda, couldn't | 0.0294 | -0.06 | -0.02 | -0.09 |
| pics, pictures, post, senior, taking, upload, posted, check, posting, camera, uploading, videos, uploaded, prom, pix | 0.0303 | -0.06 | -0.02 | -0.09 |
| read, book, reading, pages, chapter, books, written, page, article, notes, chapters, write, story, history, newspaper | 0.0338 | -0.06 | -0.02 | -0.09 |
| looked, walked, started, ran, turned, felt, gave, stood, walking, asked, sat, stopped, threw, jumped, began | 0.0369 | -0.05 | -0.02 | -0.09 |
| weeks, couple, days, months, past, left, ago, month, haven't, pregnant, moving, hasn't, stressful, havnt, grounded | 0.0369 | -0.05 | -0.02 | -0.09 |
| vacation, days, summer, mini, cruise, weeks, beach, needed, planning, enjoying, officially, florida, starts, trip, woohoo | 0.0400 | -0.05 | -0.02 | -0.09 |
| finally, found, yay, figured, months, finaly, yey, nevermind, searching, yessss, yesss | 0.0408 | -0.06 | -0.02 | -0.09 |
| books, read, book, reading, library, sees, bible, open, carry, comic, headache, satan, living, defeated, takers | 0.0416 | -0.05 | -0.02 | -0.09 |
| moving, stuff, packing, move, house, boxes, moved, place, apartment, unpacking, cleaning, furniture, pack, storage, packed | 0.0416 | -0.05 | -0.02 | -0.09 |
| tomorrow, zoo, rally, pep, assembly, prep, flags, pals, columbus, kiddos, pumped, aquarium | 0.0416 | -0.05 | -0.02 | -0.09 |
| week, work, days, rest, vacation, working, relaxation, starting, needed, begins, longest, survived, rough, recover, week's | 0.0416 | -0.05 | -0.02 | -0.09 |
| start, working, decided, finally, quit, job, legit, making, started, dang, putting, randomly, wood, decides, needa | 0.0436 | -0.05 | -0.02 | -0.09 |
| finally, yay, haircut, arrived, months, woohoo, sorted, gots, yayy, yaaay, phew, cleared, yesss, yayyyy, laptop | 0.0449 | -0.05 | -0.02 | -0.09 |
| homework, finish, finished, procrastination, finishing, due, procrastinate, assignment, studying, procrastinating, math, laundry, assignments, tons, avoiding | 0.0449 | -0.06 | -0.02 | -0.09 |
| work, yay, home, tomorrow, early, booo, woohoo, headed, tgif, payday, yuck | 0.0449 | -0.05 | -0.02 | -0.09 |
| today, yesterday, sucked, worked, luckily, rocked | 0.0466 | -0.05 | -0.02 | -0.09 |
| cookies, chocolate, making, chip, baking, brownies, cookie, bake, sugar, baked, yummy, cupcakes, oatmeal, milk, cake | 0.0470 | -0.05 | -0.02 | -0.09 |
| school, tomorrow, yay, skip, tmrw, ughh, exited, dreading, bleh, skipping, ew, yaaay, eww, ehh, orientation | 0.0488 | -0.06 | -0.02 | -0.09 |
| year, senior, freshman, junior, seniors, college, freshmen, officially, prom, orientation, highschool, skip, graduation, graduate, yearbook | 0.0488 | -0.05 | -0.02 | -0.09 |
| bowling, tonight, fun, alley, ally, blast, wii, midnight, bowl, strike, strikes, pin, league, tournament, bingo | 0.0497 | -0.05 | -0.02 | -0.09 |

**Table A3**

*All Significant High Work-to-Personal Conflict Topics*

| **Top Words** | ***p*-value** | **Effect Size** | **95% CI (lower)** | **95% CI (upper)** |
| --- | --- | --- | --- | --- |
| feels, weird, kinda, feel, feeling, wierd, bit, felt, strange, odd, hmm, suddenly, sort, awkward, dunno | 0.0278 | 0.08 | 0.04 | 0.11 |
| kinda, sad, sorta, sucks, bummed, feelin, sucked, lame, upset, depressing, pissed, tho, scary, disappointed, depressed | 0.0278 | 0.07 | 0.04 | 0.11 |

**Table A4**

*All Significant Low Work-to-Personal Conflict Topics*

| **Top Words** | ***p*-value** | **Effect Size** | **95% CI (lower)** | **95% CI (upper)** |
| --- | --- | --- | --- | --- |
| happy, birthday, wishing, sister, years, wonderful, st, daughter, nephew, brother, son, turns, niece, special, celebrate | 0.0060 | -0.09 | -0.05 | -0.12 |
| happy, birthday, wished, wishing, b-day, thankyou | 0.0060 | -0.08 | -0.05 | -0.12 |
| happy, birthday, wishing, daddy, mommy, brother, belated, dearest, advance, mama, momma, beloved, papa, returns, greet | 0.0062 | -0.08 | -0.05 | -0.12 |
| birthday, happy, wishes, b-day, celebrate, celebrating, birthdays, present, celebration, cake, celebrated, mom's, belated, dad's, brother's | 0.0062 | -0.08 | -0.05 | -0.12 |
| party, birthday, bday, b-day, surprise, celebrate, st, saturday, celebrating, bash, cake, blast, celebration, graduation, planning | 0.0062 | -0.08 | -0.04 | -0.12 |
| happy, birthday, birthdays, camper, wishing, wished, happiest, endings | 0.0231 | -0.07 | -0.04 | -0.11 |
| birthday, wishes, happy, present, wished, cheers, birthdays, celebration, belated | 0.0265 | -0.07 | -0.04 | -0.11 |
| weather, nice, loving, perfect, beautiful, enjoying, degree, enjoy, lovin, beach, lovely, gorgeous, crappy, cooler, warm | 0.0278 | -0.07 | -0.03 | -0.11 |

**Appendix B**

**All Significant Conflict LIWC Categories**

**Table B1**

*All Significant High Personal-to-Work Conflict LIWC Categories*

| **Category** | ***p*-value** | **Effect Size** | **95% CI (lower)** | **95% CI (upper)** |
| --- | --- | --- | --- | --- |
| Swear words  Netspeak  2nd person (You)  Reward | 0.0002  0.0017  0.0063  0.0353 | 0.09  0.08  0.06  0.05 | 0.05  0.04  0.03  0.01 | 0.12  0.11  0.10  0.09 |

**Table B2**

*All Significant Low Personal-to-Work Conflict LIWC Categories*

| **Category** | ***p*-value** | **Effect Size** | **95% CI (lower)** | **95% CI (upper)** |
| --- | --- | --- | --- | --- |
| Prepositions  Quantities  Articles  Home  Conjunctions  Time  Numbers  1st person plural (We)  Past focus  Motion  Auxiliary verbs  Common verbs | 1.44E-07  0.0002  0.0006  0.0007  0.0020  0.0044  0.0105  0.0205  0.0205  0.0228  0.0376  0.0394 | -0.12  -0.08  -0.08  -0.08  -0.07  -0.06  -0.06  -0.06  -0.05  -0.06  -0.05  -0.05 | -0.08  -0.05  -0.05  -0.04  -0.03  -0.03  -0.02  -0.02  -0.02  -0.02  -0.01  -0.01 | -0.15  -0.12  -0.12  -0.11  -0.11  -0.10  -0.10  -0.09  -0.09  -0.09  -0.09  -0.09 |

**Table B3**

*All Significant High Work-to-Personal Conflict LIWC Categories*

| **Category** | ***p*-value** | **Effect Size** | **95% CI (lower)** | **95% CI (upper)** |
| --- | --- | --- | --- | --- |
| Negative emotion  Fatigue  Negative tone | 0.0328  0.0328  0.0328 | 0.07  0.06  0.06 | 0.03  0.03  0.03 | 0.10  0.10  0.10 |

**Table B4**

*All Significant Low Work-to-Personal Conflict LIWC Categories*

| **Category** | ***p*-value** | **Effect Size** | **95% CI (lower)** | **95% CI (upper)** |
| --- | --- | --- | --- | --- |
| Positive tone | 0.0332 | -0.08 | -0.04 | -0.11 |

**Appendix C**

**Vocabulary Size Comparisons**

**Table C1**

*Vocabulary Size of Top 10% vs. Bottom 10% Conflict Individuals*

|  | **Predicted Personal-to-Work Conflict** | **Predicted Work-to-Personal Conflict** |
| --- | --- | --- |
| Top 10% | 24,455.30 | 32,216.95 |
| Bottom 10% | 15,534.91 | 28,887.06 |

**Table C2**

*Vocabulary Size of Top 20% vs. Bottom 20% Conflict Individuals*

|  | **Predicted Personal-to-Work Conflict** | **Predicted Work-to-Personal Conflict** |
| --- | --- | --- |
| Top 20% | 27,175.10 | 38,734.15 |
| Bottom 20% | 22,563.59 | 24,898.49 |

**Appendix D**

**Data Transparency Table**

The data reported in the current study were collected as part of the larger data collection. Findings from the data collection have been reported in a separate manuscript which has been published in a different journal.

| **Variables in the Complete Dataset** | **MS 1**  **(Status = Current paper)** | **MS 2**  **(Status = Published in a different journal)** |
| --- | --- | --- |
| Work-to-Personal Conflict | x |  |
| Personal-to-Work Conflict | x |  |
| Loneliness (ULS-3) |  | x |
| Depression (PHQ-9) |  | x |
